# The relationship between heart rate variability and eye movement characteristics in adolescents with internet gaming disorder: the mediating role of impulsivity

**DOI:** 10.3389/fpsyt.2026.1975790

**Published:** 2026-09-30

**Authors:** Yibo Zhang, Huijie Zhang, Jing Yao, Bailing Huang, Jie He, Fenglan Niu, Yanyan Li

**Affiliations:** The Fifth Clinical Medical College of Henan University of Chinese Medicine (Zhengzhou People’s Hospital), Zhengzhou, Henan, China

**Keywords:** adolescents, eye movement, heart rate variability, impulsivity, internet gaming disorder, mediating effect

## Abstract

**Background:**

This study aimed to investigate alterations in heart rate variability (HRV) and exploratory eye-movement characteristics in adolescents with Internet Gaming Disorder (IGD), examine the associations among HRV, impulsivity, and exploratory eye-movement characteristics, and further conduct an exploratory mediation analysis based on the observed correlation pattern.

**Methods:**

A cross-sectional case-control study was conducted involving 75 adolescents who met the diagnostic criteria for IGD and 75 age-, sex-, and education-matched healthy controls (HCs). All participants completed psychological assessments, including the 20-item Internet Gaming Disorder Test, the Chinese Revised Version of the Barratt Impulsiveness Scale-11 (BIS-11), the Hamilton Anxiety Scale, and the Hamilton Depression Scale. HRV parameters, including heart rate (HR), standard deviation of NN intervals (SDNN), and root mean square of successive differences (RMSSD), were collected. Eye movement parameters included the number of eye fixations (NEF), responsive search scores (RSS), total eye scanning length (TESL), mean eye scanning length (MESL), and discriminant analysis score. Statistical analyses were performed using SPSS version 25.0. Pearson correlation analyses were conducted to examine associations among variables, with Benjamini–Hochberg false discovery rate (FDR) correction applied for multiple comparisons. Mediation analyses were performed using the PROCESS macro in SPSS with the bootstrap method to test the significance of indirect effects.

**Results:**

(1) Compared with the HC group, the IGD group exhibited reduced HRV, characterized by significantly decreased RMSSD and SDNN values and increased HR (all p < 0.001). (2) Compared with the HC group, the IGD group showed significant alterations in eye movement performance, including decreased NEF and RSS, as well as reduced TESL and MESL (all p < 0.001). (3) Pearson correlation analyses within the IGD group revealed that RMSSD was negatively correlated with BIS-11 total scores, BIS-11 total scores were negatively correlated with RSS, and RMSSD was positively correlated with RSS. (4) The exploratory mediation analysis showed a significant indirect association between RMSSD and RSS through BIS-11 total scores (unstandardized indirect effect = 0.0375, 95% bootstrap CI [0.0061, 0.0903]), accounting for approximately 33.0% of the total association.

**Conclusion:**

This study examined the interrelationships among physiological, cognitive, and behavioral characteristics of IGD. Adolescents with IGD exhibited reduced HRV, increased impulsivity, and altered exploratory eye-movement characteristics. Impulsivity statistically accounted for part of the association between RMSSD and RSS.

## 1 Introduction

With the rapid development of internet technology and the widespread use of smart devices, online gaming has become one of the most common forms of entertainment among adolescents. However, excessive gaming has increasingly contributed to the emergence of Internet Gaming Disorder (IGD). IGD is characterized by excessive or uncontrolled engagement in online gaming despite long-term negative consequences, resulting in impairments in interpersonal relationships, social functioning, and physical and mental health. Meta-analytic evidence indicates that the pooled prevalence of IGD among adolescents and young adults across 33 countries is 9.9% (1). In China, a recent meta-analysis specifically focusing on adolescents reported a pooled IGD prevalence of 10% (2), and the prevalence of IGD continues to increase (3). IGD is not only closely associated with psychological problems, including anxiety (4), depression (5), and impulsivity (6), but may also lead to impaired academic functioning, disrupted interpersonal relationships, and reduced social adaptation abilities (7, 8), resulting in long-term negative consequences for individuals and their families (9). Due to the significant public health implications of IGD, the American Psychiatric Association included IGD as a condition requiring further research and clinical investigation in the Diagnostic and Statistical Manual of Mental Disorders, Fifth Edition (DSM-5) in 2013 (10). Furthermore, in 2019, the World Health Assembly approved the International Classification of Diseases, Eleventh Revision (ICD-11), officially recognizing gaming disorder (GD) as a new disorder within the category of disorders due to addictive behaviors (11). Therefore, investigating the underlying mechanisms involved in the development and maintenance of IGD is of substantial importance.

### 1.1 Heart rate variability and internet gaming disorder

At the physiological level, IGD has been associated with abnormalities in both the autonomic nervous system (ANS) (12) and the central nervous system (CNS) (9). The ANS and CNS are closely interconnected in terms of both anatomical structure and functional regulation (13). Heart rate variability (HRV) is an important physiological indicator for assessing ANS function and reflects the dynamic balance between sympathetic and parasympathetic activity (14, 15). Time-domain HRV parameters, such as the standard deviation of NN intervals (SDNN) and the root mean square of successive differences (RMSSD), are commonly used to characterize ANS functioning. SDNN represents overall HRV and reflects the flexibility of cardiac responses to external stimuli, whereas RMSSD reflects short-term variations in heart rate and is primarily considered an indicator of parasympathetic activity (16).

Previous studies have demonstrated reduced HRV in adolescents with IGD (17), characterized by increased heart rate (HR) and decreased SDNN and RMSSD values (16, 18, 19). Reduced SDNN indicates impaired HRV regulation and may reflect a sustained state of physiological stress (14). This autonomic abnormality appears to be more pronounced during gaming-related conditions. Specifically, adolescents with IGD exhibit significantly reduced high-frequency (HF) components during periods of high attentional demand, suggesting impaired parasympathetic regulation (15, 20). In addition, prolonged gaming behavior has been associated with enhanced sympathetic activity and increased low-frequency (LF)/HF ratios, indicating ANS imbalance (16, 21).

HRV is not merely a peripheral physiological marker but is also closely associated with CNS regulation, particularly neural networks involving the prefrontal cortex (PFC) (22). According to the Neurovisceral Integration Model, reduced HRV in adolescents with IGD may be associated with impaired top-down inhibitory control mediated by the PFC (14, 23), potentially contributing to difficulties in executive control and attentional regulation (24).

### 1.2 Impulsivity and internet gaming disorder

Impulsivity is considered one of the core psychological characteristics of behavioral addictions (25). Impulsivity is associated with alterations in spontaneous brain activity, particularly abnormalities in the structure and function of brain regions involved in executive control, such as the PFC, which are considered important neurobiological substrates underlying impaired impulse control (26). Impulse control impairment refers to difficulties in inhibiting engagement in certain behaviors. Previous studies have demonstrated that adolescents with IGD exhibit impaired impulse control (27), and IGD severity is positively associated with impulsivity (6, 28).

During tasks assessing impulse control, adolescents with IGD demonstrate increased impulsive decision-making tendencies. Specifically, in delay discounting tasks (DDT), adolescents with IGD are more likely to choose smaller immediate rewards rather than larger delayed rewards, indicating impaired delay gratification ability. Moreover, deficits in impulse control have been linked to dysfunctions in brain regions associated with behavioral regulation (27). Neuroimaging studies have shown that adolescents with IGD exhibit abnormal spontaneous brain activity, particularly involving the PFC (29). Given the critical role of the PFC in impulsivity regulation (30), reduced PFC activity during impulse-control-related tasks in adolescents with IGD suggests impaired neural mechanisms underlying inhibitory control (31).

### 1.3 Eye movement and internet gaming disorder

At the cognitive level, attentional bias is considered a core characteristic of addictive behaviors (32) and plays an important role in guiding addictive behaviors (33). It is also regarded as a key feature of IGD (33). Attentional bias refers to the preferential processing of addiction-related stimuli, which is closely associated with craving and the maintenance of addictive behaviors (34). Numerous studies have demonstrated the presence of attentional bias in adolescents with IGD (32, 35–37). When exposed to gaming-related visual stimuli, adolescents with IGD show enhanced attentional bias accompanied by increased neuroelectrophysiological responses, such as enhanced late positive potential (LPP) amplitudes (38).

Furthermore, in terms of temporal attentional processing, adolescents with IGD exhibit an increased attentional blink effect, suggesting difficulties in allocating attentional resources and disengaging attention from specific stimuli (33). Eye movement studies have also provided behavioral evidence of abnormalities in attentional control and visual information processing in IGD. In antisaccade tasks, adolescents with IGD demonstrate higher error rates when responding to gaming-related stimuli, indicating impaired inhibitory control. These findings suggest that attentional bias may represent a sensitive biological marker for identifying IGD as an addictive behavior (32). Moreover, high-risk adolescents with IGD show impaired inhibitory control and increased impulsivity during eye movement tasks (39).

In addition, during exploratory eye movement tasks, adolescents with IGD may exhibit abnormalities in parameters such as the number of eye fixations (NEF) and responsive search scores (RSS), suggesting differences in visual exploration efficiency and search strategies. From a neurobiological perspective, dysfunctions within the executive control network (ECN) and dorsal attention network (DAN) are considered important neural substrates underlying these behavioral alterations (6). Importantly, exploratory eye movement paradigms using non-gaming stimuli primarily characterize general visual exploration and visual search performance rather than gaming-specific attentional bias.

Although previous studies have independently demonstrated physiological and cognitive abnormalities in IGD from the perspectives of HRV, impulsivity, and eye movement, few studies have integrated these three aspects, particularly among adolescents with IGD. According to the Neurovisceral Integration Model (40), the ANS, particularly parasympathetic regulation, is closely interconnected with the PFC. Through neurophysiological mechanisms, these systems interact to regulate individual responses to external stimuli. This interaction is considered a fundamental basis for psychological and behavioral functions, including impulse control and attentional regulation. Accordingly, individual differences in HRV may be associated with impulsivity and exploratory eye-movement performance, and these variables may show interrelated patterns in adolescents with IGD.

Overall, the Neurovisceral Integration Model may provide a novel theoretical framework for understanding the physiological and cognitive mechanisms underlying IGD. Therefore, based on previous findings, the present study recruited adolescents and compared HRV parameters, including HR, SDNN, and RMSSD, as well as eye movement parameters, including the NEF, RSS, total eye scanning length (TESL), mean eye scanning length (MESL), and discriminant analysis score (D-score), between adolescents with IGD and healthy controls. Furthermore, we examined the associations among HRV, impulsivity, and exploratory eye-movement characteristics within the IGD group. Based on the observed correlation pattern, we subsequently conducted an exploratory mediation analysis to further characterize the statistical interrelationships among these variables.

## 2 Materials and methods

### 2.1 Participants and procedures

#### 2.1.1 IGD group

Adolescents with IGD who visited the Department of Clinical Psychology at Zhengzhou People’s Hospital between January 2026 and June 2026 were recruited as the IGD group. Before the study began, based on the previous clinical volume of our department and the practical feasibility of recruitment, we set a target sample size of approximately 150 participants, with approximately 75 participants planned for each group to obtain relatively balanced case and control samples. The group consisted of 75 participants, including 61 males and 14 females. The inclusion criteria were as follows: (1) meeting the diagnostic criteria for IGD according to the DSM-5, with the diagnosis independently confirmed by two psychiatrists specializing in mental disorders; (2) aged 12–18 years, of either sex, and of Han ethnicity; (3) a score of ≥71 on the 20-item Internet Gaming Disorder Test (IGD-20); (4) playing online games for ≥4 h per day or ≥30 h per week; and (5) normal vision or corrected-to-normal vision. The exclusion criteria were as follows: (1) a history of or current psychiatric disorders, including depressive disorders, anxiety disorders, schizophrenia, attention-deficit/hyperactivity disorder (ADHD), and other neurodevelopmental disorders. ADHD and other neurodevelopmental disorders were primarily assessed through clinical psychiatric evaluation conducted by psychiatrists based on the ICD-10 diagnostic criteria, together with review of participants’ previous psychiatric and medical history and collateral information obtained from their guardians regarding the adolescents’ developmental, behavioral, and psychiatric history; (2) a history of severe traumatic brain injury or neurological disorders, such as encephalitis or epilepsy; (3) significant cardiovascular diseases or cardiac arrhythmias; (4) use of any psychiatric medication or receipt of other treatments, including physical therapy or psychotherapy, within the previous month; (5) a history of substance dependence, including alcohol, nicotine, or other addictive substances; (6) severe visual or auditory impairment or inability to complete the eye movement experiment; (7) failure of eye movement calibration or inadequate data quality; and (8) unwillingness to cooperate or failure to complete the entire assessment procedure. During the recruitment period, 84 adolescents were assessed for eligibility for the IGD group. Nine were excluded: five because of depressive disorder, two because of eye-movement calibration failure, and two because of psychiatric medication use within the previous month. Thus, 75 adolescents with IGD were included in the study.

#### 2.1.2 Healthy control group

Adolescents recruited through public advertisements between January 2026 and June 2026 were included as the healthy control (HC) group. The HC group consisted of 75 participants, including 57 males and 18 females. The inclusion criteria were as follows: (1) not meeting the DSM-5 diagnostic criteria for IGD; (2) aged 12–18 years, of either sex, and of Han ethnicity; (3) an IGD-20 score <71; (4) playing online games for <4 h per day and <30 h per week; and (5) normal vision or corrected-to-normal vision. The exclusion criteria were identical to those applied in the IGD group. During the recruitment period, 78 adolescents were assessed for eligibility for the HC group. Three were excluded: two because of depressive disorder and one because of eye-movement calibration failure. Thus, 75 adolescents were included.

IGD diagnostic status was independently evaluated by two psychiatrists. Inter-rater agreement for the initial diagnostic classification was high, with an observed agreement of 96.7% and a Cohen’s κ of 0.933. In cases of initial disagreement, the final diagnostic classification was determined by consensus after discussion between the two psychiatrists.

This study strictly followed the ethical principles outlined in the Declaration of Helsinki and was approved by the Ethics Committee of Zhengzhou People’s Hospital (Ethics approval number: 2025-KY-012001). Before the study commenced, researchers provided detailed information regarding the study objectives, procedures, and potential risks to all participants and their legal guardians. Written informed consent was obtained from all participants and their families.

### 2.2 Sociodemographic data collection

General demographic information was collected from all participants, including sex, age, years of education, and daily or weekly gaming duration. Participants also underwent psychological assessments, HRV measurements, and eye movement measurements. All researchers received standardized training before study initiation to ensure the reliability and consistency of data collection.

### 2.3 Psychological assessment

To comprehensively assess IGD severity, impulsivity, and emotional symptoms, relevant psychological scales were administered. Self-report questionnaires were completed independently by participants, whereas clinician-rated scales were assessed by trained staff from the Department of Clinical Psychology.

#### 2.3.1 20-item internet gaming disorder test

The IGD-20 was used to evaluate the severity of IGD symptoms. The IGD-20 was developed by Pontes et al. (41) based on the DSM-5 diagnostic criteria for IGD and was translated and culturally adapted into Chinese by Qin Lixia et al. (42). The IGD-20 is based on the components model of addiction and assesses six dimensions: salience, mood modification, tolerance, withdrawal symptoms, conflict, and relapse. The scale consists of 20 items rated on a 5-point Likert scale (1 = “strongly disagree” to 5 = “strongly agree”), with total scores ranging from 20 to 100. A total score exceeding 71 indicates a potential diagnosis of IGD, with higher scores indicating greater IGD severity. Previous studies have demonstrated that the IGD-20 has good reliability and validity in Chinese populations (42).

#### 2.3.2 Chinese revised version of the Barratt impulsiveness scale-11

The BIS-11 was used to assess participants’ impulsivity levels. The BIS-11 was originally developed by Barratt et al. (43) and translated and revised into Chinese by Li Xianyun et al. (44). The BIS-11 consists of three subscales: non-planning impulsiveness, motor impulsiveness, and cognitive impulsiveness. The scale contains 30 items, with 10 items in each subscale. Items are rated on a 5-point scale (1 = “never” to 5 = “always”). Each subscale has a score range of 10–50. For calculation, raw scores of each subscale and the total score are converted to a 0–100 scale according to the following formula: Subscale score = [(sum of item scores − 10)/40] × 100. The total BIS-11 score is calculated as the sum of the three subscale scores divided by three. Higher scores indicate greater impulsivity. Previous studies have demonstrated good reliability and validity of the BIS-11 in Chinese populations (44).

#### 2.3.3 Hamilton anxiety scale

The Hamilton Anxiety Scale (HAMA) was used to assess the severity of anxiety symptoms. HAMA was developed by British psychiatrist Max Hamilton in 1959 and remains one of the most widely used instruments for evaluating anxiety symptoms in clinical and research settings (45). The scale evaluates two major domains: (1) somatic anxiety, including muscle tension, cardiovascular symptoms, respiratory symptoms, and gastrointestinal symptoms; and (2) psychic anxiety, including anxious mood, tension, fear, insomnia, and cognitive symptoms. The HAMA consists of 14 items rated on a 5-point scale (0 = “absent” to 4 = “very severe”), with total scores ranging from 0 to 56. Higher scores indicate more severe anxiety symptoms.

#### 2.3.4 Hamilton depression scale

The Hamilton Depression Scale (HAMD) was used to assess the severity of depressive symptoms. HAMD was developed by British psychiatrist Max Hamilton in 1960 and is one of the most widely applied clinician-rated instruments for assessing depressive symptoms (46). The HAMD has commonly used 17-item and 24-item versions. In the present study, the 17-item version was adopted, covering affective, somatic, and cognitive symptoms. The assessment includes: (1) affective symptoms, such as depressed mood, guilt, and suicidal ideation; (2) somatic symptoms, including insomnia, appetite reduction, weight loss, and physical discomfort; and (3) cognitive symptoms, including impaired attention, psychomotor retardation, and feelings of worthlessness. Higher total scores indicate more severe depressive symptoms.

### 2.4 HRV measurement

Resting-state HRV was assessed using the CM400 heart rate variability analysis system. In the present study, photoplethysmographic (PPG) signals were acquired using a finger-mounted optical pulse sensor, with a sampling frequency of ≥500 Hz. The system was equipped with real-time signal monitoring, automatic artifact detection, and correction functions. All participants were assessed in the same supine position in a quiet, temperature-controlled environment. To reduce potential physiological variability, pre-measurement conditions were standardized with respect to recent physical exercise, food intake, sleep, and time of day. After a 5-min resting adaptation period, continuous beat-to-beat interval data were recorded for 5 min.

Time-domain parameters were selected for statistical analysis, including: (1) HR, defined as the number of heartbeats per minute (bpm); (2) SDNN, reflecting overall beat-to-beat variability during the recording period; and (3) RMSSD, representing the root mean square of successive differences between adjacent beat-to-beat intervals and primarily reflecting short-term vagally mediated variability. HRV data analysis followed international HRV measurement standards (47) to ensure reliability and comparability.

### 2.5 Eye movement measurement

An exploratory eye movement (EEM) paradigm was used to assess eye movement characteristics. Eye movement data were collected using the DEM-2000B eye movement detection system (Shanghai Dikon Medical Biotechnology Co., Ltd.). The EEM testing procedure was based on the protocol reported by Kojima et al. (48). The system uses infrared pupil-tracking technology with a non-contact measurement approach. It has a sampling rate of ≥120 Hz, spatial measurement accuracy of ±0.10° (reading precision of 0.01°), and pupil diameter measurement accuracy of ±0.10 mm, allowing simultaneous binocular tracking and highly stable recording. Experiments were conducted under stable lighting conditions with minimal environmental interference. Before testing, participants underwent standardized 9-point calibration. Participants were seated comfortably, maintaining a distance of approximately 25–30 cm between their eyes and the display screen, with both eyes horizontally aligned with the center of the screen. During the experiment, three “S-shaped” images (S1, S2, and S3) were presented sequentially, with each image displayed for 15 s. Participants first observed the S1 image, followed by the S2 and S3 images, which contained slight differences from the original image. After viewing all three images, participants were asked to indicate verbally whether they had noticed any differences between S2/S3 and S1 and to briefly describe the differences they had noticed. These verbal responses were not scored and were not included in the statistical analyses.

Eye movement parameters were automatically extracted by the system, including: (1) NEF: the number of fixation points exceeding 200 ms during the 15-s image viewing period; (2) RSS: S2 and S3 images were divided into seven regions, and the number of different regions fixated within 5 s was recorded, with a maximum total score of 14; (3) TESL: reflecting the overall scanning range of eye movements; (4) MESL: reflecting the average distance of individual saccades; (5) D-score: a composite score mathematically derived from NEF and RSS according to the formula D = 10.265 − (0.065 × NEF + 0.871 × RSS). Because the D-score is mathematically determined by NEF and RSS rather than independently measured, it was treated in the present study only as an exploratory descriptive composite index and not as an independent eye-movement parameter. NEF reflects the extent of visual exploration in terms of the number of fixations, RSS reflects visual search performance for altered regions of the stimulus, TESL characterizes the overall extent of visual scanning, and MESL reflects the average scanning distance between successive eye movements. These parameters reflect participants’ attentional maintenance ability, visual search ability, and cognitive processing efficiency from multiple perspectives (49). In the digital eye movement recording system, detected eye movements were automatically analyzed using a computerized EEM analysis system (50–52).

### 2.6 Statistical analysis

Statistical analyses were performed using SPSS version 25.0. The Shapiro–Wilk test was used to assess the normality of continuous variables, and homogeneity of variance between groups was evaluated using Levene’s test. Categorical variables were compared using chi-square tests. Normally distributed continuous variables were expressed as mean ± standard deviation (Mean ± SD), and between-group comparisons were conducted using independent-samples t-tests. Correlation and mediation analyses were conducted within the IGD group. Pearson correlation analyses were first performed to explore associations among HRV parameters, BIS-11 scores, and exploratory eye-movement variables. To account for multiple testing, the Benjamini–Hochberg false discovery rate (FDR) procedure was applied, with an FDR-adjusted p value (q value) < 0.05 considered statistically significant. The mediation model was not prespecified *a priori*. Rather, after inspection of the correlation matrix, RMSSD, BIS-11, and RSS showed a consistent pattern of statistically significant pairwise associations. Therefore, a subsequent exploratory mediation analysis was conducted to further characterize the statistical relationship among these three variables. RMSSD was entered as the predictor, BIS-11 total score as the statistical mediator, and RSS as the outcome in Model 4 of the PROCESS macro in SPSS (53). The mediation models were estimated using variables in their original measurement scales. Unstandardized regression coefficients (B), standard errors (SE), t values, and p values were reported for statistical inference, together with standardized regression coefficients (β) to facilitate comparison across variables. The bootstrap method was applied with 5,000 bootstrap samples and 95% confidence intervals (CIs) to evaluate indirect effects. A mediation effect was considered significant when the 95% CI did not include zero. The proportion of the indirect effect relative to the total effect was calculated to evaluate the magnitude of the mediation effect. A two-tailed *p* value < 0.05 was considered statistically significant.

## 3 Results

### 3.1 Comparison of sociodemographic characteristics between the two groups

There were no statistically significant differences between the IGD group and the HC group in terms of age, years of education, or sex (*p* > 0.05), and these comparisons remained nonsignificant after FDR correction (all q > 0.05). The detailed demographic characteristics are presented in **Table 1**.

**Table 1:** Sociodemographic characteristics of participants in the two groups.

Variables	IGD group (n=75)	HC group (n=75)	t/χ2	P
Age(x ± s)^a^	14.91 ± 1.96	14.83 ± 1.82	0.259	0.796
Years of education(x ± s)^a^	8.91 ± 2.07	8.88 ± 1.85	0.083	0.934
Sex(male/female)^b^	61/14	57/18	0.636	0.425
Weekly gaming duration^a^	41.64 ± 8.66	9.84 ± 5.37	27.023	<0.001

^a^independent-samples t-test; ^b^chi-square test; IGD, Internet Gaming Disorder; HC, healthy control.

### 3.2 Comparison of psychological scale scores between the two groups

Compared with the HC group, the IGD group exhibited significantly higher scores on the IGD-20 and BIS-11 (*p* < 0.001), indicating greater severity of IGD symptoms and higher levels of impulsivity. In addition, the IGD group showed significantly higher scores on the HAMA and HAMD compared with the HC group (*p* < 0.001), suggesting increased anxiety and depressive symptoms among adolescents with IGD. All reported psychological-scale differences remained statistically significant after FDR correction. The detailed results are presented in **Table 2**.

**Table 2:** Comparison of psychological scale scores between the two groups.

Variables		IGD group	HC group	t	P
IGD20		80.49 ± 6.71	37.80 ± 5.51	42.576	<0.001
	Salience	11.71 ± 1.34	5.81 ± 1.34	26.872	<0.001
	Mood Modification	12.07 ± 1.57	5.96 ± 1.60	23.602	<0.001
	Tolerance	12.05 ± 1.41	5.71 ± 1.33	28.290	<0.001
	Withdrawal	12.12 ± 1.38	4.89 ± 1.03	36.210	<0.001
	Conflict	20.37 ± 2.51	9.92 ± 2.21	27.046	<0.001
	Relapse	12.17 ± 1.52	5.51 ± 1.35	28.415	<0.001
BIS-11		62.22 ± 7.04	48.30 ± 6.09	12.947	<0.001
	Cognitive	62.36 ± 8.52	48.25 ± 6.96	11.109	<0.001
	Motor	60.50 ± 7.45	45.95 ± 6.53	12.712	<0.001
	Non-planning	68.24 ± 9.24	50.71 ± 7.55	12.722	<0.001
HAMA		13.41 ± 1.77	7.92 ± 2.09	17.360	<0.001
HAMD		14.29 ± 1.81	7.79 ± 2.15	20.056	<0.001

IGD-20, 20-item internet gaming disorder test; BIS-11, Barratt Impulsiveness Scale 11^th^; HAMA, Hamilton anxiety scale; HAMD, Hamilton depression scale; IGD, internet gaming disorder; HC, healthy control.

### 3.3 Comparison of HRV parameters between the two groups

Compared with the HC group, adolescents in the IGD group exhibited significantly decreased HRV, characterized by significantly lower RMSSD and SDNN values (*p* < 0.001), whereas HR was significantly increased (*p* < 0.001). All three between-group differences remained statistically significant after FDR correction, suggesting reduced vagal modulation and reduced overall autonomic flexibility in adolescents with IGD. The detailed results are presented in **Table 3**.

**Table 3:** Comparison of HRV parameters between the two groups.

Variables	IGD group	HC group	95% CI	Cohen’s d	t	P
HR	74.73 ± 4.84	72.12 ± 4.62	[1.09, 4.14]	0.55	3.382	<0.001
SDNN	40.40 ± 13.44	51.92 ± 13.00	[−15.79, −7.25]	−0.87	−5.336	<0.001
RMSSD	36.73 ± 7.04	48.48 ± 6.43	[−13.93, −9.58]	−1.74	−10.678	<0.001

HR, Heart Rate; SDNN, Standard Deviation of NN intervals; RMSSD, Root Mean Square of Successive Differences; IGD, internet gaming disorder; HC, healthy control.

### 3.4 Comparison of eye movement parameters between the two groups

Compared with the HC group, the IGD group exhibited significant differences in eye movement parameters. Specifically, the IGD group showed significantly lower NEF and RSS values (*p* < 0.001), as well as significantly reduced TESL and MESL values (*p* < 0.001). All of these between-group differences remained statistically significant after FDR correction. These findings suggest decreased visual exploration efficiency and altered attentional allocation patterns in adolescents with IGD. The detailed results are presented in **Table 4**.

**Table 4:** Comparison of eye movement parameters between the IGD and HC groups.

Variables	IGD group	HC group	95% CI	Cohen’s d	t	P
NEF	23.73 ± 3.10	29.87 ± 2.08	[−6.99, −5.28]	−2.32	−14.228	<0.001
RSS	6.55 ± 1.67	9.87 ± 1.00	[−3.76, −2.88]	−2.41	−14.748	<0.001
TESL	214.31 ± 31.65	260.88 ± 31.91	[−56.83, −36.32]	−1.47	−8.974	<0.001
MESL	8.72 ± 1.08	9.84 ± 1.04	[−1.46, −0.78]	−1.06	−6.492	<0.001
D-score	2.61 ± 1.28	-0.25 ± 0.91				

NEF, number of eye fixations; RSS, responsive search scores; TESL, total eye scanning length; MESL, mean eye scanning length; IGD, internet gaming disorder; HC, healthy control.

### 3.5 Exploratory correlation analysis of HRV, BIS-11, and exploratory eye-movement parameters in the IGD group

Exploratory Pearson correlation analyses were conducted within the IGD group to examine associations among HRV parameters, BIS-11 scores, and exploratory eye-movement parameters. After Benjamini–Hochberg FDR correction for 21 pairwise correlation tests, RMSSD remained negatively associated with BIS-11 scores (r = −0.429, q < 0.001), BIS-11 scores remained negatively associated with RSS (r = −0.471, q < 0.001), and RMSSD remained positively associated with RSS (r = 0.441, q < 0.001). Given this consistent pattern of FDR-corrected associations among RMSSD, BIS-11, and RSS, these three variables were subsequently examined in an exploratory mediation analysis. The detailed results are presented in **Table 5**.

**Table 5:** Correlation analysis of HRV, BIS-11, and eye movement parameters in the IGD group.

Variables	SDNN	RMSSD	BIS-11	NEF	RSS	TESL	MESL
SDNN	1						
RMSSD	0.322**	1					
BIS-11	-0.186	-0.429**	1				
NEF	0.032	0.095	-0.220	1			
RSS	0.250	0.441**	-0.471**	0.419**	1		
TESL	0.217	0.198	-0.273*	0.297**	0.276*	1	
MESL	0.219	0.212	-0.213	0.149	0.287*	0.298**	1

P values were adjusted using the Benjamini–Hochberg FDR procedure. FDR-adjusted p values (q values) < 0.05 were considered statistically significant. *q < 0.05; **q < 0.01; SDNN, Standard Deviation of NN intervals; RMSSD, Root Mean Square of Successive Differences; BIS-11, Chinese Revised Version of the Barratt Impulsiveness Scale-11; NEF, number of eye fixations; RSS, responsive search scores; TESL, total eye scanning length; MESL, mean eye scanning length.

### 3.6 Exploratory mediation analysis of the association between RMSSD and RSS through BIS-11

Based on the observed pattern of pairwise correlations, an exploratory mediation analysis was performed to examine whether BIS-11 total scores statistically accounted for part of the association between RMSSD and RSS in the IGD group. In the mediation model, RMSSD was entered as the independent variable, BIS-11 total score as the mediator, and RSS as the dependent variable. Considering that sex (54), age (55), anxiety symptoms (4), and depressive symptoms (5) may influence IGD-related characteristics, sex and age were included to account for demographic and developmental variation, whereas HAMA and HAMD scores were included to account for individual differences in anxiety and depressive symptoms, which are commonly associated with IGD-related characteristics. These variables (sex, age, HAMA, and HAMD scores) were included as covariates in the mediation model. Given the cross-sectional design, the mediation analysis was interpreted as a statistical decomposition of associations rather than evidence of temporal precedence or a causal mechanism. The specification of RMSSD as the predictor, BIS-11 total score as the mediator, and RSS as the outcome reflected the analytic model and did not establish the temporal or causal ordering of these variables.

In Model 1, RMSSD was significantly and positively associated with RSS (β = 0.479, *p* < 0.001). In Model 2, RMSSD was significantly and negatively associated with BIS-11 total scores (β = −0.413, *p* < 0.001). In Model 3, after including BIS-11 in the statistical mediation model, the association between RMSSD and RSS remained significant (β = 0.321, *p* < 0.01), while BIS-11 total scores were significantly and negatively associated with RSS (β = −0.383, *p* < 0.001). These findings indicate a significant indirect association between RMSSD and RSS through BIS-11 total scores, with impulsivity statistically accounting for part of the association between RMSSD and RSS. The detailed results are presented in **Table 6**.

**Table 6:** Exploratory mediation analysis of the association between RMSSD and RSS through BIS-11.

Model	Result variables	Prediction variables	B	SE	β	t	p
Model 1	RSS	RMSSD	0.114	0.025	0.479	4.644	<0.001
Model 2	BIS-11	RMSSD	−0.413	0.110	−0.413	-3.764	<0.001
Model 3	RSS	RMSSD	0.076	0.025	0.321	3.080	0.003
	RSS	BIS-11	−0.091	0.025	−0.383	-3.676	<0.001

B, unstandardized regression coefficient; SE, standard error of the unstandardized coefficient; β, standardized regression coefficient; RMSSD, Root Mean Square of Successive Differences; BIS-11, Chinese Revised Version of the Barratt Impulsiveness Scale-11; RSS, responsive search scores.

### 3.7 Effect size analysis of the mediation model

In the unstandardized effect decomposition, the total association between RMSSD and RSS was 0.114 (95% CI [0.065, 0.163]), and the direct association after including BIS-11 was 0.076 (95% CI [0.027, 0.126]). The bootstrap indirect association through BIS-11 was 0.038 (BootSE = 0.022, 95% bootstrap CI [0.006, 0.090]). The indirect association accounted for approximately 33.0% of the total association. The mediation effect was considered significant because the 95% CI of the indirect effect did not include zero. The detailed results are presented in **Table 7** and **Figure 1**.

**Table 7:** Unstandardized total, direct, and indirect associations between RMSSD and RSS through BIS-11.

Effect	Effect value	BootSE	LLCI	ULCI	Effect ratio
Direct effect(c′) RMSSD → RSS	0.076	0.025	0.027	0.126	67.0%
Indirect effect(a*b) RMSSD → BIS-11 → RSS	0.038	0.022	0.006	0.090	33.0%
Total effect(c)	0.114	0.025	0.065	0.163	100%

LLCI, Lower Limit Confidence Interval; ULCI, Upper Limit Confidence Interval; RMSSD, Root Mean Square of Successive Differences; BIS-11, Chinese Revised Version of the Barratt Impulsiveness Scale-11; RSS, responsive search scores.

**Figure 1:**
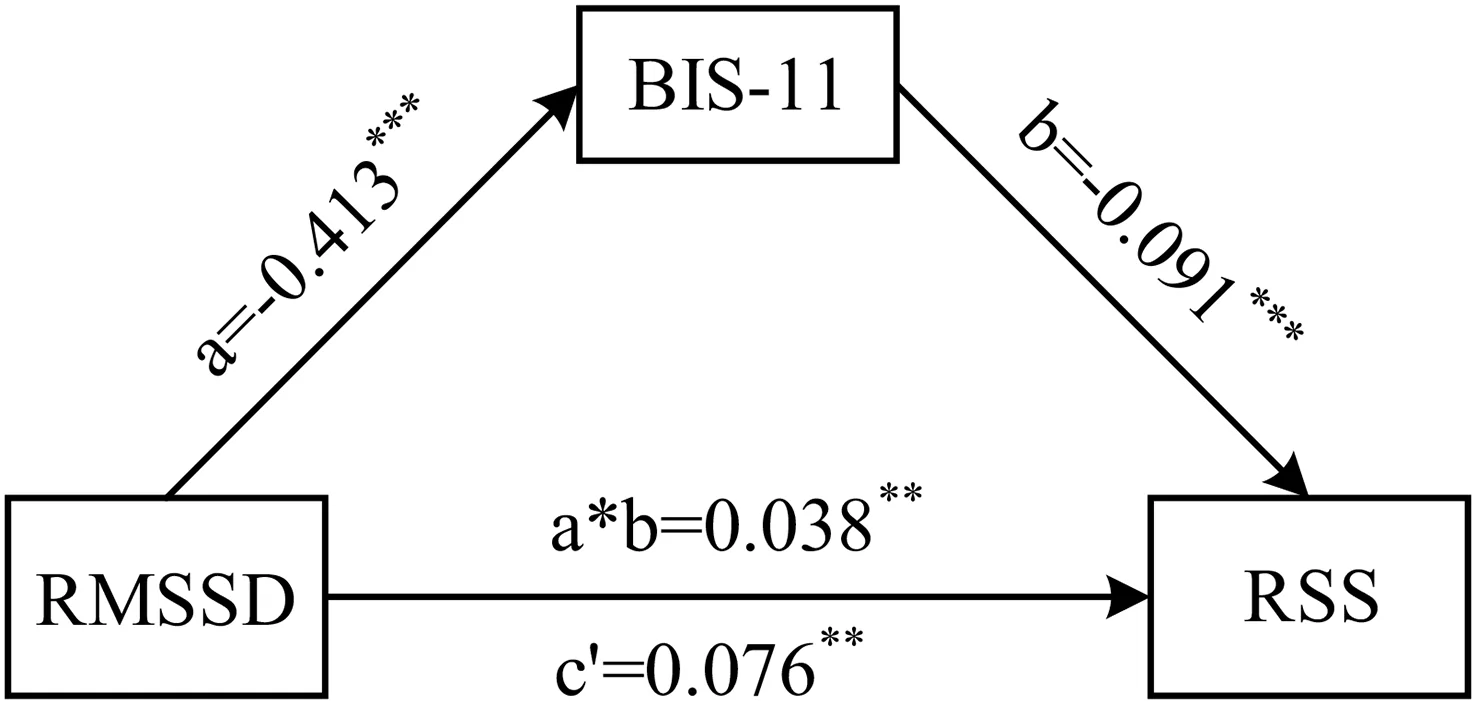
Exploratory mediation model of the association between RMSSD and RSS through BIS-11(a*b, indirect effect; c’, direct effect; ***p < 0.001; **p < 0.01; RMSSD, Root mean square of successive differences; BIS-11, Chinese revised version of the Barratt impulsiveness scale-11; RSS, responsive search scores).

## 4 Discussion

The present study integrated HRV and eye movement measures to investigate the potential mechanisms underlying IGD from physiological, cognitive, and behavioral perspectives. The results demonstrated that: (1) regarding HRV parameters, adolescents with IGD exhibited reduced HRV, characterized by significantly decreased RMSSD and SDNN, along with increased HR; (2) regarding eye movement parameters, adolescents with IGD showed altered eye movement patterns, characterized by significantly lower NEF and RSS, as well as reduced TESL and MESL, indicating decreased visual exploration efficiency and abnormal attentional allocation patterns; and (3) within the IGD group, significant associations were observed among HRV, impulsivity, and eye movement parameters, with impulsivity statistically accounting for part of the association between RMSSD and RSS.

### 4.1 HRV abnormalities in adolescents with IGD

The present study demonstrated reduced HRV in adolescents with IGD, which is consistent with previous findings (16–19). From a physiological perspective, HRV is considered an important indicator of ANS flexibility and adaptive capacity. Among HRV parameters, RMSSD primarily reflects parasympathetic (vagal) regulation, whereas SDNN represents overall heart rate variability and the integrated regulatory capacity of the ANS (56). The concurrent reductions in RMSSD and SDNN observed in the present study suggest reduced short-term vagal modulation and lower overall autonomic flexibility in adolescents with IGD. According to the Neurovisceral Integration Model (40), HRV reflects the functional connectivity between the ANS and the PFC (22). Reduced HRV has been associated with impaired PFC regulatory capacity, suggesting deficits in emotional regulation, impulse control, and attentional regulation (57, 58). In the context of IGD, diminished autonomic regulation may increase adolescents’ susceptibility to game-related reward cues, thereby enhancing sensitivity to immediate rewards and weakening delayed gratification and behavioral inhibition abilities (59).

Furthermore, previous studies suggest that prolonged exposure to high-intensity gaming stimuli may be associated with altered autonomic regulation and reduced physiological recovery following heightened arousal (15). Such altered autonomic regulation may be associated with difficulties in emotional regulation and reduced efficiency of cognitive control processes relevant to addictive behaviors (14). Therefore, the present study provides important evidence for understanding the physiological basis of IGD and offers a theoretical foundation for future interventions targeting autonomic regulation, such as HRV biofeedback training (60).

### 4.2 Eye movement characteristics abnormalities in adolescents with IGD

The present study found that, compared with healthy controls, adolescents with IGD exhibited significant alterations in exploratory eye-movement characteristics, primarily characterized by reduced visual exploration efficiency and altered visual search patterns. From a cognitive perspective, eye movement behaviors provide objective behavioral information regarding visual exploration, attentional allocation, and cognitive control processes during information processing (32). The observed alterations in eye movement suggest that adolescents with IGD may exhibit a more restricted pattern of visual exploration and reduced visual search efficiency. This pattern may indicate differences in the allocation of visual attention and less efficient exploration of complex visual information.

Previous studies using gaming-related stimuli have demonstrated attentional bias toward gaming-related cues in adolescents with IGD (33, 35). In these gaming-cue paradigms, attentional bias may involve enhanced attentional capture and difficulty disengaging attention from gaming-related stimuli, resulting in preferential allocation of attentional resources toward gaming-related information. However, the exploratory eye movement paradigm used in the present study employed non-gaming S-shaped images and did not include a comparison between gaming-related and neutral stimuli. Therefore, the present findings should be interpreted as reflecting exploratory eye-movement characteristics, including visual exploration and visual search performance, rather than gaming-specific attentional bias.

From a neurobiological perspective, eye movement parameters can serve as objective behavioral indicators of visual exploration and attentional processing. These processes are closely associated with PFC function (32, 61) and involve multiple neural networks. Previous research has demonstrated altered functional connectivity between the ECN and the DAN in adolescents with IGD (6). Dysfunction within these networks may contribute to impaired cognitive control and disrupted top-down attentional allocation. Therefore, the altered exploratory eye-movement characteristics observed in adolescents with IGD provide further behavioral evidence of differences in visual exploration and attentional processing.

### 4.3 Exploratory statistical mediation of the association between RMSSD and RSS by impulsivity

The exploratory mediation analysis indicated that impulsivity statistically accounted for part of the association between RMSSD and RSS. According to the Neurovisceral Integration Model (40), HRV has been associated with the functional integration of autonomic regulation and prefrontal cortical processes involved in self-regulation (23). Lower HRV has also been associated with poorer executive functioning. The higher impulsivity observed in adolescents with IGD may be consistent with alterations in executive control processes; however, the present cross-sectional data do not establish the direction of this association. Previous research has linked impaired inhibitory control and preference for immediate gratification with persistent gaming behavior despite negative consequences (62). Furthermore, impaired executive function may compromise attentional regulation and visual information processing (63). These alterations may be associated with exploratory eye-movement characteristics, including differences in attentional allocation and reduced visual search efficiency (32, 33, 64).

These findings may be interpreted within the theoretical framework of the I-PACE model (Interaction of Person-Affect-Cognition-Execution model) (65). The I-PACE model proposes that addictive behaviors emerge from the interaction among individual predispositions (Person), affective states (Affect), cognitive processes (Cognition), and executive functions (Execution). Within this framework, HRV, impulsivity, and exploratory eye-movement characteristics may represent interrelated physiological, psychological, and behavioral features associated with IGD. The significant indirect association observed in the present statistical model is compatible with this theoretical framework, but it does not establish that changes in HRV temporally precede or causally influence impulsivity or eye movement performance.

Taken together, the present findings demonstrate statistical associations among HRV, impulsivity, and exploratory eye-movement characteristics in adolescents with IGD. The observed indirect association is consistent with a potential interplay among autonomic regulation, executive control, and visual information processing, although the temporal ordering of these variables remains to be established.

### 4.4 Limitations

Several limitations of the present study should be acknowledged. First, because all variables were assessed within a cross-sectional framework, the statistical mediation analysis cannot establish temporal precedence or causal relationships among RMSSD, impulsivity, and RSS. The direction specified in the mediation model represents an analytic and theory-guided specification rather than an empirically established temporal sequence. Alternative explanations, including reverse or bidirectional associations and shared underlying factors affecting all three variables, cannot be excluded. Longitudinal and experimental studies are therefore needed to clarify the temporal ordering and potential causal relationships among these variables. Second, no *a priori* power analysis was conducted specifically for the exploratory mediation analysis. Although the observed indirect association was supported by 5,000-sample bootstrap estimation, the sample of 75 adolescents with IGD limits the precision and generalizability of the mediation estimates. Therefore, the mediation findings should be considered exploratory and require replication in larger independent samples. Third, HRV measurements in this study were conducted only under resting conditions. Although resting-state HRV can reflect baseline autonomic regulation, it cannot capture the dynamic changes in ANS regulation in adolescents with IGD when exposed to gaming-related cues. Future research should incorporate task-based HRV assessments to further investigate the relationship between autonomic function and IGD. Finally, the exploratory eye movement paradigm used in the present study employed non-gaming visual stimuli and therefore assessed general exploratory eye-movement characteristics, including visual exploration and visual search performance, rather than gaming-specific attentional bias. In addition, eye movement parameters alone cannot reveal the underlying neurocognitive mechanisms. Future studies should incorporate paradigms that directly compare gaming-related and neutral stimuli and combine eye movement measures with functional magnetic resonance imaging (fMRI) to further investigate the neural mechanisms underlying altered attentional processing in IGD.

## 5 Conclusions

In conclusion, this study integrated HRV, impulsivity, and exploratory eye-movement measures to investigate their associations in adolescents with IGD from physiological, psychological, and behavioral perspectives. The findings demonstrated that adolescents with IGD exhibited reduced HRV, increased impulsivity, and altered exploratory eye-movement characteristics. Moreover, impulsivity statistically accounted for part of the association between RMSSD and RSS. Within the framework of the Neurovisceral Integration Model and the I-PACE model, these findings provide preliminary evidence for the interrelationships among autonomic regulation, impulsivity, and exploratory eye-movement performance in adolescents with IGD. Given the cross-sectional design, the temporal ordering and causal relationships among these variables cannot be determined.

## Data Availability

The original contributions presented in the study are included in the article/supplementary material. Further inquiries can be directed to the corresponding author.
